# Analysis of Epistasis among QTLs on Heading Date based on Single Segment Substitution Lines in Rice

**DOI:** 10.1038/s41598-018-20690-w

**Published:** 2018-02-15

**Authors:** Zifeng Yang, Lingling Jin, Haitao Zhu, Shaokui Wang, Guiquan Zhang, Guifu Liu

**Affiliations:** 1 Guangdong Key Lab of Plant Molecular Breeding, College of Agronomy, South China Agricultural University, Guangzhou, 510642 P. R. China; 2 College of Mathematics and Informatics, College of Software Engineering, South China Agricultural University, Guangzhou, 510642 P. R. China

## Abstract

Heading date directly determines the planting districts and seasons, and thus plays an important role for producing and introducing of varieties. Limited to the materials and methodologies, analysis of epistasis still presents an obvious challenge. This thesis estimated effectively four types of epistatic components among dual QTLs on heading date based on eight single segment substitution lines (SSSLs) in rice. The results confirmed that they carried truly with heading date QTLs. Eleven pairs of QTLs were with 50.0% of significant epistatic effects, of which additive-additive, additive-dominance or dominance-additive, and dominance-dominance interaction components occupied 40.9%, 50.0% and 59.1%, respectively. One QTL always interacted with multiple QTLs in various components. Several characteristics of epistasis on heading date were found that 1) different epistatic components had almost consistent directions; 2) dominance-dominance epistasis was perhaps most important in the four epistatic components; 3) epistasis was mostly positive, delaying rice heading; and 4) all epistatic components were seasonal sensitive. Two flowering pathways were further confirmed via a network constructed among these QTLs. These results have further confirmed the prevalence of epistatic interactions, deepened the understanding of genetic and flowering mechanism, and excavated several advantageous genes on heading date in rice.

## Introduction

The concept “breeding by design” has become an international advantageous technology to guide genetic improvement and breeding for crops and the basic strategy to culture green super rice in China^1^. The preconditions for design breeding are to identify the locations and to fully comprehend the functions of QTLs on various important agronomic traits, and to possess materials with available genes^2^. Our lab had constructed a platform including 1529 single segment substitution lines (SSSLs)^3,4^ to explore fine genes and then to design breeding. Using these SSSLs we had identified lots of QTLs and assessed their allelic variations on some important agronomic traits^5–7^. Recently, we attempted to pyramid several interesting QTLs underlying one trait or varying traits into an elite variety by the molecular marker technology^8^. However, the efficiency and process of breeding by design were limited by the lack of enough understanding for epistatic interactions among QTLs manipulated.

Epistasis was defined as the interactions among non-alleles on a genome. It is one of important genetic components for complex quantitative traits, which typical genetic characteristics included interactions among non-alleles and between genes and environments^9^. Epistatic effects between loci were suggested to estimate as the deviation of the genotypic effect from the sum of all single locus effects underlying the trait based on the linear additive model^10^. Japanese rice genome projects (JRGP) identified at least 15 QTLs on heading date in rice and analyzed their epistatic interactions via developing series of near isogenic lines (NILs)^11,12^. Our lab using SSSLs identified and analyzed a mass of QTLs and their epistatic interactions on some major agronomic traits^13,14^.

Heading date is a critical agronomic trait, which directly determines the adaptation to specific cropping locations and growing seasons for current varieties of cultivated rice, and thus plays an important role for producing and introducing of rice varieties. Heading date in rice is also a complex quantitative trait, determined by a multiple QTL system companying with additive, dominance and epistasis, as well as their interaction with environments. There were two independent flowering pathways to control heading time in rice. One was the conserved *Hd1*-dependent pathway and the other unique *Ehd1*-dependent^15,16^. *Hd1* controlled flowering through regulating *Hd3a*^17^, while *Ehd1* promoted flowering by activating *Hd3a* and *RFT1* (rice flowering locus T1)^15,16^. However, the genetic interactions among these factors are still not well understood.

Previous studies few effectively estimated various epistatic components on heading date QTLs in rice simultaneously, such as additive-additive, additive-dominance or dominance-additive, and dominance-dominance epistatic effects for dual QTLs. However they play respectively different roles in evolution system and speciation, inbreeding depression and heterosis, genetic architecture of complex traits and development of new varieties^18^. In this study, we used eight SSSLs as experimental materials to estimate additive and dominant effects of six QTLs (*Hd1*, *Ehd1*, *Hd3a* or *RFT1*, *EH3*, *OsMADS50* and *DTH8*) and their epistatic effects among dual QTLs. The aims were to further confirm the prevalence of epistatic interactions, to deeply understand genetic and flowering mechanism and to excavate useful alleles for molecular pyramiding breeding and heterosis breeding on heading date in rice.

## Results

### Variations of heading date and analysis of variance (ANOVA)

The frequency distributions of heading date in rice for F_2_ populations with two QTLs were continuous and approximately normally distributed (Supplementary Fig. 1 and Supplementary Fig. 2). In theory, there are only nine genotypes for each F_2_ populations of two QTLs, according with the separation ratio of genotypes (1AA:2Aa:1aa) (1BB:2Bb:1bb), which is the basis of normal distribution for phenotypes. However, these distributions were of skewness frequently due to the influence from interactions of the alleles, non-allelic genes or other environmental factors.

In order to clarify the causes of the variation of heading date, we carried out ANOVA, according to the statistical model *y*_*hij*_ = *μ* + *E*_*h*_ + *G*_*i*_ + (*GE*)_*hi*_ + *B*_*j*(*h*)_ + *e*_*hij*_ (where *E*_*h*_, *G*_*i*_ and (*GE*)_*hi*_ were the *h*th environmental effect, the *i*th genotypic effect and their interaction effect, while *y*, *μ*, *B* and *e* were phenotypic value, population mean, the *j*th block effect in the *h*th environment and the residual, respectively) for the heading date at the both seasons of 2014 (Supplementary Table 1). The ANOVA showed that the main factors of the variations of heading date were environments, genotypes and their interactions. Genetically, heading date was different with different genotypes and/or different environments.

**Table 1:** The eight single segment substitution lines (SSSLs) and their basic information.

SSSL	Code	Chr.	Marker on substitution segment	Donor parent	Putative QTL
W23-03-08-9-27-82	*S*_*1*_	3	Terminal–PSM301-PSM304–RM569	Lemont	*OsMADS*5*0*
W05-01-11-02-07-06 (A)	*S*_*2*_	3	Terminal–RM569-RM232–RM282	Zihui100	*EH3*
W08-18-09-09-06-02	*S*_*3*_	6	RM549-RM136-RM527	IR64	*Hd1*
W04-47-68-05-04-04-02-02	*S*_*4*_	6	RM510–RM204-RM50–RM549	BG367	*Hd3a or RFT1*
W05-01-11-02-07-06 (B)	*S*_*5*_	8	RM22468–RM22475-RM5432–RM22490	Zihui100	*DTH8-1*
W06-26-35-01-05-02	*S*_*6*_	8	PSM152–PSM154-RM72–RM404	Katy	*DTH8-2*
W11-17-03-07-05-08	*S*_*7*_	10	PSM166–RM596-RM271–RM269	Basmati 370	*Ehd1-1*
W27-18-03-21	*S*_*8*_	10	RM467–PSM166-RM304–RM294A	IAPAR9	*Ehd*1*-*2

**Table 2:** A half diallel crossing population constructed from four parents. Numbers 0, 1 and 2 represented genotypes *aa, Aa* and *AA*, respectively.

			
			
			
			

### QTL effects of SSSLs

The previous research showed that these SSSLs used in this experiment all carried with heading date QTLs in rice. The effects of these SSSLs were also estimated under the early and the late season of 2014, respectively, further confirming that the eight SSSLs truly took along heading date QTLs (Table 3).

**Table 3:** Additives and dominances of the eight SSSLs estimated on heading date in the both seasons of 2014 (day).

SSSL	QTL effect	Estimation	
		The early season	The late season
*S*_*1*_	*a*	−17.61^**^	−9.23^**^
	*d*	−18.80^**^	−10.02^**^
*S*_*2*_	*a*	−1.15	−0.99
	*d*	−1.20	−1.68^*^
*S*_*3*_	*a*	−0.47	0.74
	*d*	−3.67^**^	0.86
*S*_*4*_	*a*	22.76^**^	19.84^**^
	*d*	3.73^**^	6.59^**^
*S*_*5*_	*a*	18.47^**^	8.51^**^
	*d*	13.67^**^	4.82^**^
*S*_*6*_	*a*	17.75^**^	4.60^**^
	*d*	8.20^**^	4.19^**^
*S*_*7*_	*a*	4.68^**^	5.68^**^
	*d*	−1.14	2.55^**^
*S*_*8*_	*a*	3.34^**^	0.44
	*d*	−0.91	0.69

SSSL was the abbreviation of single segment substitution line. *S*_*i*_ represented the code of SSSL_i_. *a* and *d* were additive and dominant effect, respectively. Sign “-” meant the early heading alleles from donors. Superscripts “^*^ and ^**^” indicated the significances at 5% and 1% level, respectively.

The eight SSSLs were detected with significant additives and/or dominances in the two cropping seasons. Since SSSLs *S*_*2*_*, S*_*3*_ and *S*_*8*_ had small effects, their estimations were significant only under one of seasons, the early season or the late season. It suggested that *S*_*1*_*, S*_*2*_ and *S*_*3*_ seemed to carry with early heading genes on substitution segments. Four SSSLs *S*_*1*_, *S*_*4*_, *S*_*5*_ and *S*_*6*_ had large QTL effects, which changed greatly heading date. QTLs on *S*_*2*,_ *S*_*4*_, *S*_*7*_ and *S*_*8*_ had similar effects between the both seasons, suggesting that they were environmental-stable. Large differences between the two seasons occurred on QTLs on *S*_*1*_*, S*_*3*_, *S*_*5*_ and *S*_*6*_, implying that the four QTLs were environmental-sensitive. Three QTLs on *S*_*1*_*, S*_*2*_ and *S*_*3*_, had larger dominances than additives, which were expectable to be used to heterosis breeding.

### Epistatic interactions among QTLs

Epistatic effect was estimated as the deviation between dual-QTL pyramiding effect and sum of single-QTL effects. This study estimated epistatic effects of eleven pairs of QTLs on heading date in rice, which included four interaction components such as additive-additive (*aa*), additive-dominance (*ad*), dominance-additive (*da*) and dominance-dominance (*dd*) (Table 4).

**Table 4:** Epistatic effects estimated between QTLs on heading date in the two cropping seasons of 2014 (day).

SSSL combination	The early season				The late season			
	*aa*	*ad*	*da*	*dd*	*aa*	*ad*	*da*	*dd*
*S*_*1*_ */S*_*3*_	2.28	11.84**	8.43**	11.98**	1.17	3.36^**^	5.66^**^	5.63^**^
*S*_*1*_ */S*_*4*_	0.02	−1.22	−5.09**	4.05**	−0.29	−1.46	2.03	3.34^**^
*S*_*1*_ */S*_*5*_	12.56**	19.04**	4.82**	11.64**	3.79^**^	7.17^**^	−1.11	5.28^**^
*S*_*1*_ */S*_*6*_	0.93	4.97**	4.01**	12.74**	3.64^**^	0.87	7.45^**^	5.42^**^
*S*_*1*_ */S*_*8*_	5.47**	2.46	4.22**	5.80**	1.37	0.64	4.54^**^	4.27^**^
*S*_*2*_ */S*_*5*_	−0.48	−3.41*	−0.16	−0.28	−4.54^**^	−1.85	−1.06	0.77
*S*_*3*_ */S*_*4*_	−8.42**	−5.82**	−1.44	1.50	−1.95	−1.90	1.76	−2.02
*S*_*3*_ */S*_*6*_	1.29	4.86**	−1.14	15.25**	0.94	0.13	−1.34	−1.66
*S*_*3*_ */S*_*8*_	1.10	4.60**	6.51**	8.54**	0.14	0.96	−0.06	−1.32
*S*_*4*_ */S*_*7*_	−14.74**	2.87	−17.41**	2.00	−7.47^**^	−1.04	−15.06^**^	−0.80
*S*_*6*_ */S*_*7*_	0.04	4.21**	3.73*	7.01**	−2.22^*^	−1.62	−1.97	−1.38

SSSL was the abbreviation of single segment substitution line. *S*_*i*_ represented the code of SSSL_*i*_. *aa*, *ad*, *da* and *dd* were the additive-additive, additive-dominance, dominance-additive and dominance-dominance epistatic effects, respectively. Sign “-” indicated to improve flowering. Superscripts “^*^ and ^**^” indicated the significances at 5% and 1% level, respectively.

All of the eleven pairs of QTLs were detected with significant epistatic effects, further confirming the prevalence of epistatic interactions among QTLs. Of eighty-eight estimations, 50% of epistatic effects reached to significant level of 5%. 40.9%, 50.0% and 59.1% of *aa, ad* or *da*, and *dd* epistatic interactions were significant, respectively. The ratios were close to the results found by Eshed and Zamir^19^ in tomato. Additionally, one QTL always interacted with multiple QTLs in various ways of components.

Several characteristics of epistasis were detected. For different epistatic components, their effect directions were almost consistent with an exception of *S*_1_*/S*_4_. Only in the early of 2014, opposite effect directions appeared between dominance-additive and dominance-dominance epistatic interactions on *S*_1_*/S*_4_. Direction consistent epistatic components reflected perhaps the common feature of epistasis on target trait. Between the two cropping seasons, there was largely disparity of effect values on epistatic components, implying that they were environmental sensitive.

For the epistatic components, the effect values $(\bar{x}\pm sd)$ were 3.40 ± 0.87 day (for *aa*), 4.21 ± 0.66 day (for *ad* or *da*) and 5.12 ± 0.94 day (for *dd*), respectively, from which we could see that there were the largest and unstable estimation appeared on dominance-dominance epistasis. The larger and more stable estimation was additive-dominance or dominance-additive epistasis. The additive-additive epistasis was the smallest but unstable. The results suggested that dominance-dominance epistasis was perhaps the most important one in the four epistatic components.

Different QTL combinations produced different epistasis. The estimated values of epistasis were mostly positive, indicating that they delayed rice heading. Some negative epistatic effects promoted flowering inversely. Theoretically, epistatic effect values were inversed to sum of two QTL effects. Thus interactions between two QTLs with negative effects produced likely positive epistasis, while interactions between positive effect QTLs resulted mostly in negative epistasis. However, in fact the directions of epistatic interactions were seriously dependent on the candidate QTLs. Here, QTLs on *S*_1_, *S*_3_, *S*_6_ and *S*_8_ caused mostly positive interactions, while the others produced mainly negative epistasis. The result indicated that genes interacted according to the model themselves. On magnitude, the epistatic effects changed heading date from 3.4 days to 19.0 days in the early season, while from 3.3 days to 15.1 days in the late season.

Epistatic effects were environmental sensitive, and the length of natural sunlight changed significantly the estimations. In Table 4, significant differences on epistatic components estimated occurred on the both seasons. For example, *ad* epistasis of combination *S*_1_/*S*_3_ was estimated by 11.84 days in the early season, while it was 3.36 days in the late season. Lin *et al*.^11^ used the ranges of QTL effects between environments to illustrate the degree of environmental sensitivity (DES) of QTLs. In this paper the DES could also been used to illustrate the degree of seasonal sensitivity of QTL epistatic interactions. The DES $(\bar{x}\pm sd)$ calculated for the four epistatic components were 0.50 ± 4.66 (*aa*), 3.56 ± 4.43 (*ad*), 0.51 ± 4.41 (*da*) and 5.70 ± 5.05 (*dd*) days in turn. This result indicated that *aa/da* and *dd* had the least and largest DES values, respectively. A common finding was that they all had large standard deviations, showing that epistatic effects for some combinations were seasonal sensitive. Additionally, most of QTL epistatic effects delayed heading in the early season with some exceptions.

### A network among QTLs on heading date

**Figure 1:**
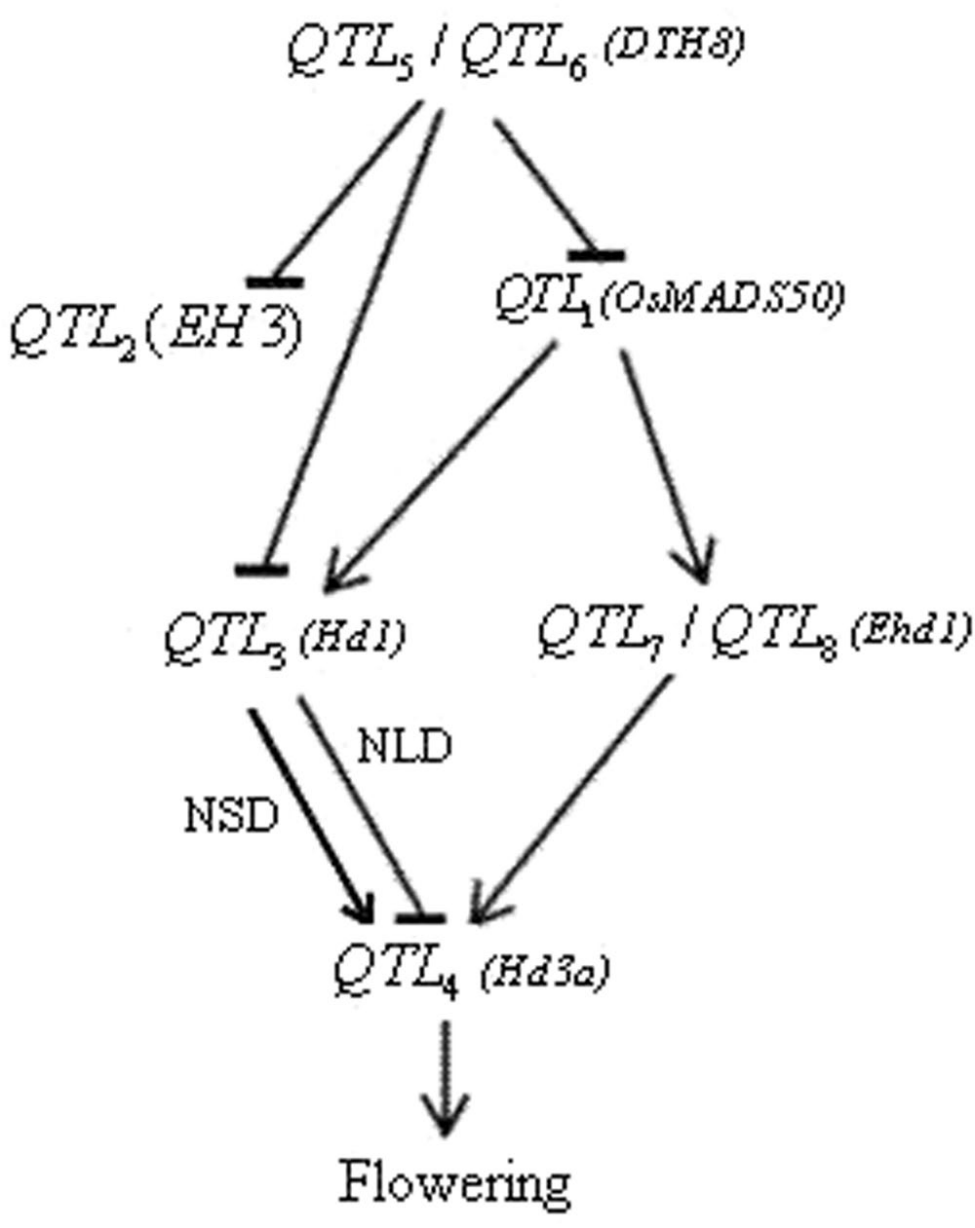
A preliminary network of rice flowering among six QTLs investigated. *QTL*_*i*_ represented QTL on *SSSL*_*i*_. *QTL*_*i*_ → *QTL*_*j*_ and *QTL*_*i*_  ⊣ *QTL*_*j*_ indicated that *QTL*_*i*_ activated and repressed the expression of *QTL*_*j*_, respectively. NLD and NSD were the abbreviations of natural long-day and short-day, respectively.

According to the genetic effect values of two SSSLs *S*_*i*_ and *S*_*j*_ (Table 3) and their polymerization lines *S*_*i*_*/S*_*j*_ (Supplementary Table 2), we could infer to the regulate relationship of two QTLs. When the value of *S*_*i*_*/S*_*j*_ was coincident with that of *S*_*i*_, it was showed that the expression of *QTL*_*j*_ was repressed by *QTL*_*i*_ (Here, *QTL*_*i*_ *or QTL*_*j*_ referred to the QTL on SSSL *S*_*i*_ or *S*_*j*_ respectively)^11,17^. To investigate the relationship between these two genes, we generated the regulating network, showed in Fig. 1.

We could see that there were two main flowering pathways control heading time in rice by regulating *QTL*_4_ expression. One was the *QTL*_3_*-*mediated pathway, in which *QTL*_3_ acted as an inducer under the natural short-day (NSD) and an inhibitor under the natural long-day (NLD) of *QTL*_4_. The other was the *QTL*_7_*/QTL*_8_*-*mediated, which took on a flowering activator of *QTL*_4_. *QTL*_5_*/QTL*_6_ as the inhibitors functioned as *QTL*_1_, *QTL*_2_ and *QTL*_3_ of the downstream. *QTL*_1_ could simultaneously promote the expressing of *QTL*_*5*_ and *QTL*_*7*_*/QTL*_*8*_ of the downstream. These results indicated a preliminary network of rice flowering among QTLs investigated, which basically were consistent with those in previous studies^15,16,20^.

### Correlations between heading date and other yield-associated traits

It was obvious that heading date associated with other yield-associated traits in rice. The coefficient of correlation was estimated as $R=\frac{CO{V}_{xy}}{{\sigma }_{x}.{\sigma }_{y}}$, where *σ*_*x*_, *σ*_*y*_ and cov_*xy*_ respectively represent standard deviation of two traits *x* and *y* as well as co-variance between them (Table 5). In the early and the late season of 2014, the result showed that there were significant phenotypic correlations between heading date and one of traits such as *ph, pl, pgn, pd*. Moreover, the significant phenotypic correlations were consistent with their genetic correlations, showing that they were mainly caused by genotypic effects. The coefficients of correlation between *hd* and *ph, pl, pd* were great different between the both seasons, suggesting that their correlations were environmental sensitive. Inversely, correlations between *hd* and *pgn, sp* were environmental stable.

**Table 5:** Correlation coefficients estimated between heading date and another related trait in the both seasons of 2014.

Type of correlation	Season	Trait pair								
		*hd-ph*	*hd-pn*	*hd-pl*	*hd-pd*	*hd-pgn*	*hd-fgn*	*hd-sp*	*hd-tkw*	*hd-gy*
Phenotype	The early	−0.089	0.019	0.667^**^	0.078	0.373^**^	0.036	−0.512^**^	0.244	0.072
	The late	0.281^**^	−0.045	0.125	0.306^**^	0.381^**^	0.074	−0.483^**^	−0.087	0.021
Genotype	The early	−0.096	0.023	0.733^**^	0.119	0.471^*^	0.056	−0.649^**^	0.264	0.100
	The late	0.375^**^	−0.071	0.146	0.362^**^	0.487^**^	0.114	−0.550^**^	−0.102	0.038
Additive	The early	0.932	0.906	0.997^**^	0.079	0.577	0.612	−0.860	−0.235	0.366
	The late	0.395	−0.530	0.110	0.542	0.678^*^	−0.273	−0.856^**^	−0.090	−0.282
Dominance	The early	−0.591	−0.002	0.814	0.044	0.435	0.037	−0.564	0.945	0.102
	The late	0.055	−0.519	0.294	−0.226	−0.143	−0.634	0.161	0.226	−0.684^*^
Epistasis	The early	0.377	0.142	0.034	0.113	0.088	0.067	−0.401	−0.445	−0.012
	The late	−0.460^**^	−0.396^**^	−0.266	0.249	0.220	−0.225	0.049	−0.186	−0.331^*^

*hd, ph, pn, pl, pd, pgn, fgn, sp, tkw* and *gy* represented heading date (days), plant height (cm), panicle number, panicle length (cm), panicle density (grain/cm), per panicle grain number, filled grain number, setting percentage (%), thousand kernel weight (g) and grain yield per plant (g), respectively. Sign “-” indicated inverse correlation between trait pairs. Superscripts “^*^ and ^**^” indicated the significances at 5% and 1% level, respectively.

Genetic component correlations contributed to most of relative genetic correlations. However, their contributions were different. Genetic correlations between *hd* and *pl, pgn, sp* derived mainly from additive effects, between *hd* and *gy* from dominant effects, while between *hd* and *ph, pn, gy* from epistatic effects. So we could see phenotypic correlations were mainly caused by genetic correlations then by different genetic component correlations on heading date.

## Discussion

Polygenic inheritance system is a complex network structure, in which the expression of genes are regulated by other genes, and in the result, epistasis is inevitable^21^. Besides understanding the biological function of single QTL, illustration of genetic interactions among these QTLs is also important. Eshed and Zamir^19^ reported that there were 20–48% of the 45 dichromosome fragment combinations were dominance by dominance epistasis on five yield related traits in tomato. Actually, some reports suggested that both additive and additive × additive interaction could explain about 73% of the total spikelets per panicle phenotypic variance^22^, and that 66 plant height QTLs tested interactions, about 42.4% were epistatic (P < 5%)^14^. There were some studies of epistatic interactions among heading date QTLs in rice to have been reported also^11,12,23–25^. Our lab also carried out extensive researches about the QTL epistasis on heading date in rice^26,27^. We found the frequency of QTL epistasis was high on heading date^28^. In this paper, 50% of epistatic effects arrived to significant level of 5%, along with 40.9%, 50.0% and 59.1% of significant additive-additive, additive-dominance or dominance-additive, and dominance-dominance epistatic components. These results further confirmed the prevalence of epistatic interactions among QTLs on heading date in rice.

Since traditional method to analysis of quantitative traits didn’t distinguish the effect of individual gene, it could only estimate epistasis mixed from multi-gene system^29^. QTL mapping methods based on bi-parental populations couldn’t provide precise estimation of epistatic effects since the interference of genetic background^30^. Using near-isogenic lines or single segment substitution lines, some epistatic components between dual QTLs were estimated^6,26^. However, previous studies few estimated simultaneously various epistatic components. Author ever constructed several secondary F_2_ populations derived from crossing of two SSSLs, each of which pyramided dual QTLs to allow simultaneously analysis of four epistatic components^28^. Nevertheless, it is time-consuming, expensive and difficult to look for all of nine genotypes from a F_2_ population via molecular marker assisted selection. As a reasonable improvement, double QTL polymerization line was developed first, and then a half diallel crossing population from four parents (receptor, two SSSLs and their DSSL) was constituted to generate nine genotypes (Table 2). This method eased to get target genotypes, lowered the cost of molecular marker analysis, and could be constructed repeatedly. Analyzing the genetic effects of the nine genotypes enabled to simultaneously estimate various epistatic components. In this paper, four epistatic components of eleven pair QTLs on heading date were successfully estimated. A mark advantage of this method is easy to extend to analyze epistasis among multiple QTLs.

In this study, we revealed the relationships among 6 heading date QTLs (Fig. 1). There were two independent flowering pathways to control heading date in rice. One was the conserved *Hd*1-dependent pathway. *Hd1* controlled flowering through regulating *Hd3a* under NLD conditions and facilitated *Hd3a* under NSD, but it was repressed and activated by the up-steam QTLs *DTH8* and *OsMADS50*, respectively. The other was unique *Ehd1*-dependent pathway. *Ehd1* promoted flowering by activating *Hd3a*. Recent researches demonstrated that the expression of *Ehd1* was promoted by a number of positive regulators as *OsMADS50* etc and was repressed by *DTH8* etc^31^. Our network showed also this relationship. *Hd3a* was down regulated by *OsMADS50*, but it is possible that an additional factor may be needed for the induction^32^. Our findings indicated it was *Hd1* or *Ehd1* that acted as the inducer. The pathway from *EH3* to *Hd3a* was still in puzzle. These results were basically consistent with those found in previous researches^15,16,20^. To improve the network of flowering, we are exploring epistatic interactions among multiple QTLs on heading date in rice.

Quantitative trait locus (QTL) mapping researches in the last few decades have identified more than 734 QTLs for heading date in rice (http://archive.gramene.org/qtl/). Some heading date QTLs in rice were aggregated by design breeding. However, early empirical studies were less successful^33^, which could not reach anticipated goals because of the existing of QTL interactions. Utilization of the known major QTLs for heading date in breeding through gene pyramiding needs take epistasis into consideration^27^. Similar studies have been reported in heading date or plant height^25,34^. Therefore, it is first important duty to understand QTL epistasis, which was directly related to the success of molecular aggregation breeding. Generally, when epistatic effect was no significant or with the same direction with the effects of constituted QTLs, this QTL combination might be considered as gene materials for molecular aggregation breeding^28^. In present study, the combinations *Hd1*/*Ehd1-*2, *EH*3/*DTH8-1*, *Hd1*/*DTH8-2*, *DTH8-2*/*Ehd1-1* and *Hd3a*/*Hd1* basically accorded with the afore-mentioned conditions, thus they were expected to reach the pyramiding aim. For example, the additives of *Hd3a* and *Hd1* delayed heading by 19.84 days and 0.74 days respectively with 1.76 days of interaction effect (Tables 3, 4), thus their pyramiding effects expected to reach 22.34 days delaying heading.

## Methods

### Plant materials

There were eight single segment substitution lines (SSSLs) and theirs receptor parent Hua-jing-xian 74 (HJX74) being selected as experimental materials in this study (Table 1). HJX74 is an elite *indica* variety, which was developed by our laboratory, with many excellent properties. Each of the eight SSSLs had possessed only single segment substituted from one donor into HJX74 genetic background^3,4^, and had been confirmed to harbor QTL/gene with significant effects on heading date^28^. By molecular markers, the data suggested that *S*_*1*_*, S*_*2*_, *S*_*3*_, *S*_*4*_, *S*_*5*_, *S*_*6*_, *S*_*7*_ and *S*_*8*_ harbor *OsMADS50*, *EHd3*, *Hd1, Hd3a or RFT1, DTH8-1*, *DTH8-2*, *Ehd1-1* and *Ehd1-2*, respectively. In the late season of 2012, eleven crosses *SSSL*_*i*_ × *SSSL*_*j*_ were made (subscripts *i* and *j* represented the serial numbers of SSSLs). Genotyping was conducted to substantiate that the F_1_ plants were not from self-pollination. Selfed-seeds of all the F_1_ plants of a cross combination were harvested and mixed to develop the F_2_ populations in the early season of 2013, which were applied to select homozygous materials pyramiding two target segments assisted by molecular markers. In the late season of 2013, HJX74 (*00*), the eight SSSLs (*02* or *20*) and their homozygous polymerization lines (*22*) were crossed each other to have constructed multiple 4 × 3/2 half diallel crossing populations (Table 2).

### Field experiments

The phenotypic experiment was conducted at the experimental station in South China Agricultural University, Guangzhou, China (23°79′ N, 113°159′ E). All materials including receptor HJX74, SSSLs, dual segment substitution lines (DSSLs), and their crossing combinations of *HJX*74 × *SSSL*_*i*_, *HJX*74 × *SSSL*_*j*_, *HJX*74 × *DSSL*_*ij*_, *SSSL*_*i*_ × *DSSL*_*ij*_, *SSSL*_*j*_ × *DSSL*_*ij*_, were simultaneously grown in the both seasons of 2014, the early season (duration from March to July, suggested as natural long-day condition, NLD) and the late season (duration from August to December, suggested as natural short-day condition, NSD). Meteorological data showed that the average duration of possible sunshine is larger than 13 hours under the early season and less than 12 hours under the late season in Guangzhou. Germinated seeds were sowed in a seedling bed, and then seedlings were transplanted to a rice field 20 days later with one plant per hill, according to the density of 16.7 cm × 16.7 cm. A completely randomized block design was adopted, in which each plot consisted of four rows with ten plants each row. The testing performed accordance management of the field with local standard practices. The heading date of twenty plants at the center of each plot was measured as the number of days from sowing to the appearance of the first panicle. Averages on heading date over twenty plants each plot were as inputting data for statistical analysis.

### Statistical analysis and estimation of QTL effects

Statistical model *y*_*ij*_ = *μ *+ *G*_*i*_ + *B*_*j*_ + *e*_*ij*_ was used to conduct analysis of variance (ANOVA) on data of all materials investigated in single season, where *y*, *μ*, *G*, *B* and *e* were the observation value each plot, population mean value, genotypic effect, block effect and the residual error, respectively. The subscripts *i* and *j* represented the serial numbers of genotypes and blocks, respectively. To confirm the existence of individual QTL, we estimated the additive effect (*a*) by (*SSSL*_*i*_−*HJX*74) and the dominant effect (*d*) by (*HJX*74 × *SSSL*_*i*_ − *HJX*74), respectively. Their significances at *α* probability level were tested by the least significant difference (*LSD*) method with the statistics $LS{D}_{\alpha }={t}_{\alpha }\sqrt{\frac{2{{S}_{e}}^{2}}{n}}$(where ${{S}_{e}}^{2}$ was the variance from experimental error, *n* was the numbers of block, and *t*_*α*_ was the critical *t*-value under *α* probability level and error freedom degree. To evaluate epistatic interactions between pairs of QTLs, the effect value (*e*) was estimated by (*DSSL*_*ij*_ + *HJX*74 − *SSSL*_*i*_ − *SSSL*_*j*_) for additive-additive epistasis (*aa*), (*SSSL*_*i*_ × *DSSL*_*ij*_ + *HJX*74 − *SSSL*_*i*_ − *HJX*74 × *SSSL*_*j*_) for additive-dominance epistasis (*ad*), (*SSSL*_*j*_ × *DSSL*_*ij*_ + *HJX*74 − *HJX*74 × *SSSL*_*i*_ − *SSSL*_*j*_) for dominance-additive epistasis (*da*), and (*HJX*74 × *DSSL*_*ij*_ + *HJX*74 − *HJX*74 × *SSSL*_*i*_ − *HJX*74 × *SSSL*_*j*_) for dominance-dominance epistasis (*dd*), which was tested by $LS{D}_{\alpha }={t}_{\alpha }\sqrt{\frac{4{{S}_{e}}^{2}}{n}}$. ANOVA and estimations of QTL effects were carried out with aov() and lm() functions in R language (https://www.r-project.org/).

## Electronic supplementary material

Supplementary Information
