# Supplementary material for: Analysis of Epistasis among QTLs on Heading Date based on Single Segment Substitution Lines in Rice
Source: Sci Rep. 2018 Feb 15;8:3059. doi: 10.1038/s41598-018-20690-w (PMC5814450; doi:10.1038/s41598-018-20690-w)
Supplement: Supplementary file 1 — Supplementary Information [file 41598_2018_20690_MOESM1_ESM.pdf]

Analysis of Epistasis among QTLs on Heading Date based on Single Segment Substitution Lines in Rice

Zifeng Yang[1#], Lingling Jin[2#], Haitao Zhu[1], Shaokui Wang[1*], Guiquan Zhang[1*], Guifu Liu[1*]

[1] Guangdong Key Lab of Plant Molecular Breeding, College of Agronomy, South China Agricultural University, Guangzhou 510642, P. R. China

[2] College of Mathematics and Informatics, South China Agricultural University, Guangzhou 510642, P. R. China

[*] Co-corresponding authors: Shaokui Wang, Guiquan Zhang and Guifu Liu

[#] Zifeng Yang and Lingling Jin contributed equally to this work.

Phone: +86–20–85281933

Fax: +86–20–85288311

E-mail: guifuliu@scau.edu.cn

Supplementary Table 1 Analysis of variance on heading date in the both cropping seasons of 2014. Superscripts “* and **” indicated the significances at 5% and 1% level, respectively.

Source of variation	Degree of freedom	Sum of squares	Mean square	F-value	P-value
Environment	1	80449.20	80449.20	62066.24**	1.028E-293
Genotype	60	25732.63	428.88	330.88**	3.310E-202
Genotype×Environment	60	5159.32	85.99	66.34**	8.207E-121
Block	2	4.31	2.15	1.66	0.19
Residual error	242	313.68	1.30	-	-

Supplementary Table 2 The genetic effect values of pyramiding materials based on genotypic values of HJX74 in the both seasons of 2014 (day). SSSL was the abbreviation of single segment substitution line. S_i represented the code of $SSSL_i$. *AABB*, *AABb*, *AaBB* and *AaBb* were putative genotypes of SSSL combination. Sign "-" indicated to improve flowering. Superscripts "* and **" indicated the significances at 5% and 1% level, respectively.

SSSL combination	The early season				The late season			
	AABB	*AABb*	*AaBB*	*AaBb*	*AABB*	*AABb*	*AaBB*	*AaBb*
S_1/S_3	-9.44**	-15.8**	-10.49**	-10.84**	-4.81**	-7.37**	-3.44**	-3.63**
S_1/S_4	-15.09**	5.17**	-11.02**	-1.14	-3.87**	10.32**	0.27	11.85**
S_1/S_5	4.48**	13.42**	6.50**	15.10**	-2.61**	3.07**	0.56	2.76**
S_1/S_6	-4.44**	1.07	2.13*	2.96**	-4.22**	-1.09	-0.41	1.97*
S_1/S_8	-16.06**	-8.80**	-13.91	-11.25**	-7.87**	-7.43**	-5.09**	-5.03**
S_2/S_5	9.12**	16.85**	12.18**	17.10**	2.02*	2.97**	4.02**	5.78**
S_3/S_4	1.83*	13.87**	1.57*	13.27**	5.39**	18.60**	5.52**	22.37**
S_3/S_6	12.59**	18.58**	19.78**	12.95**	5.06**	6.15**	3.22**	4.15**
S_3/S_8	3.22**	3.97**	3.97**	6.18**	2.39*	1.33	0.24	1.37
S_4/S_7	11.28**	12.70**	4.60**	4.21**	11.29**	18.02**	8.42**	7.38**
S_6/S_7	20.82**	22.47**	14.1**	16.61**	5.53**	8.05**	5.04**	8.03**

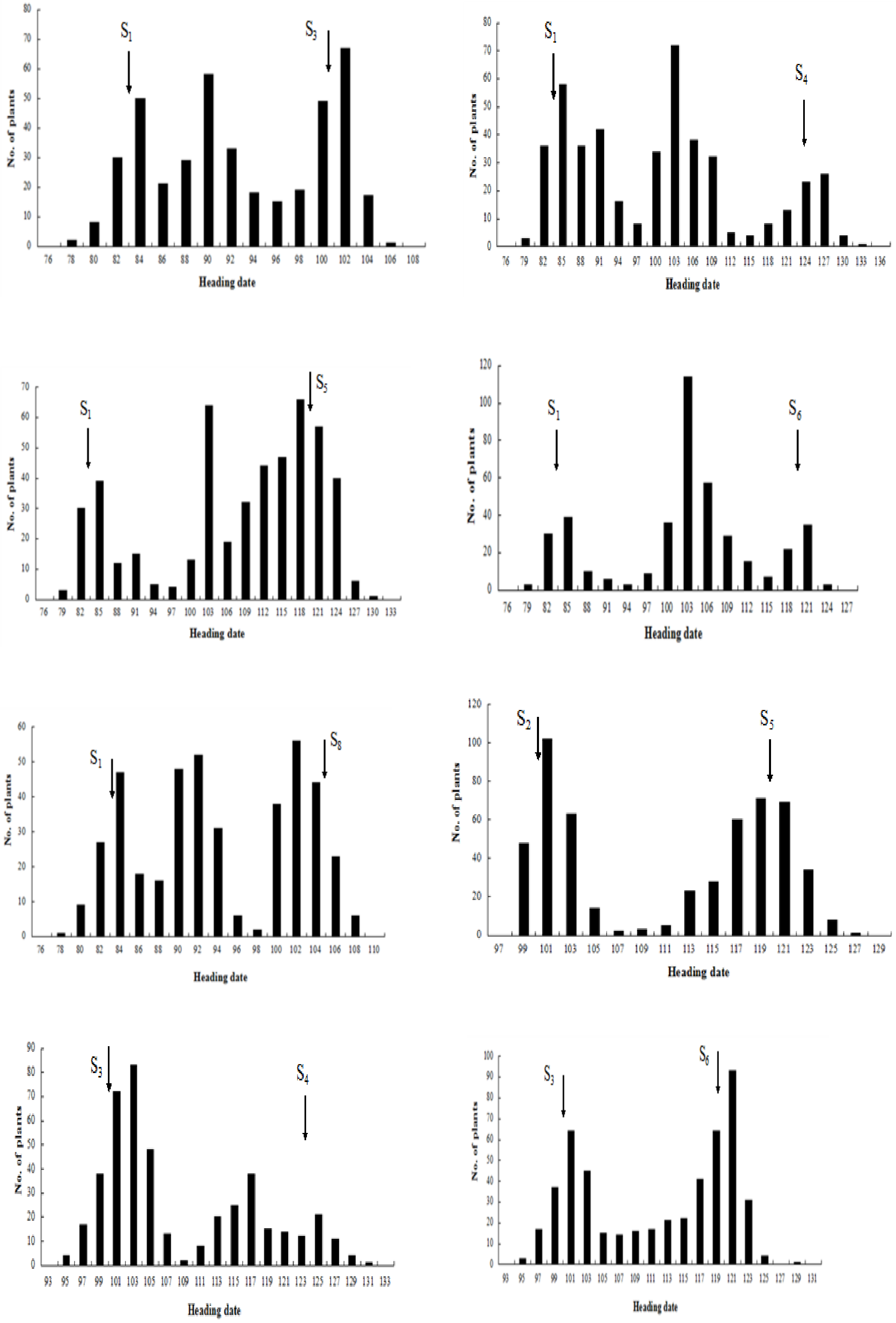
S1
S3
No. of plants
Heading date
S1
S4
No. of plants
Heading date
S1
S5
No. of plants
Heading date
S1
S6
No. of plants
Heading date
S1
S8
No. of plants
Heading date
S2
S5
No. of plants
Heading date
S3
S4
No. of plants
Heading date
S3
S6
No. of plants
Heading date

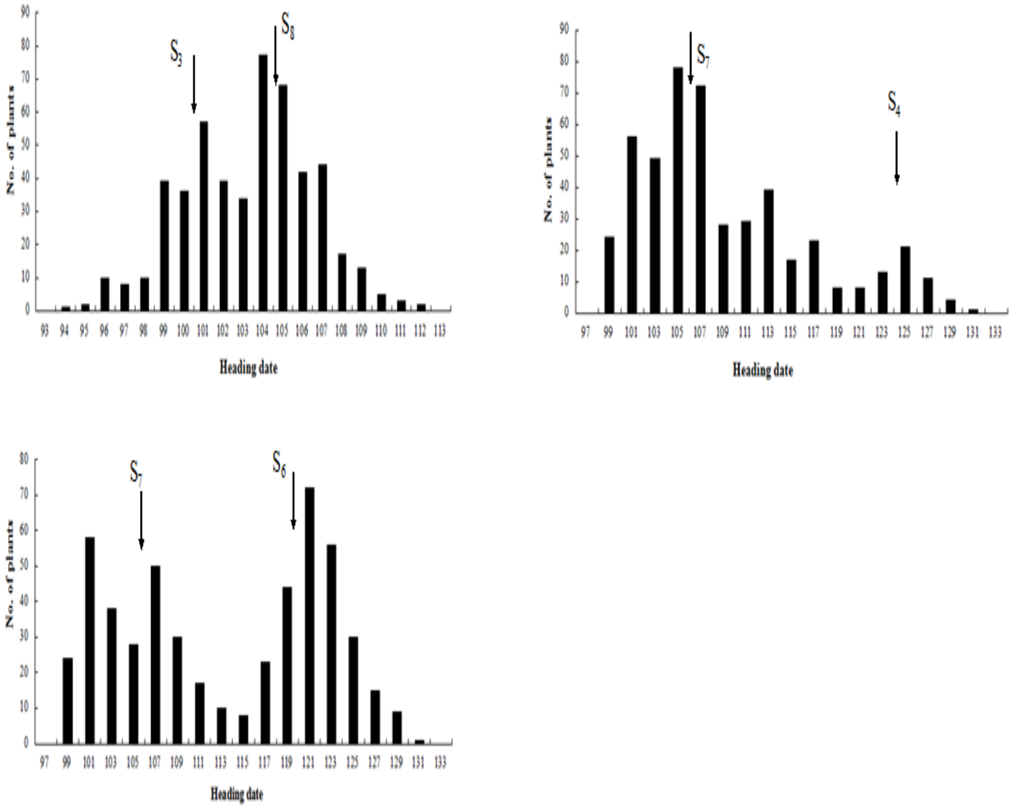

Supplementary Fig. 1 Heading date frequency distributions of F_2 populations from two single segment substitution lines in the early season of 2014. S_i represented single segment substitution line $SSSL_i$

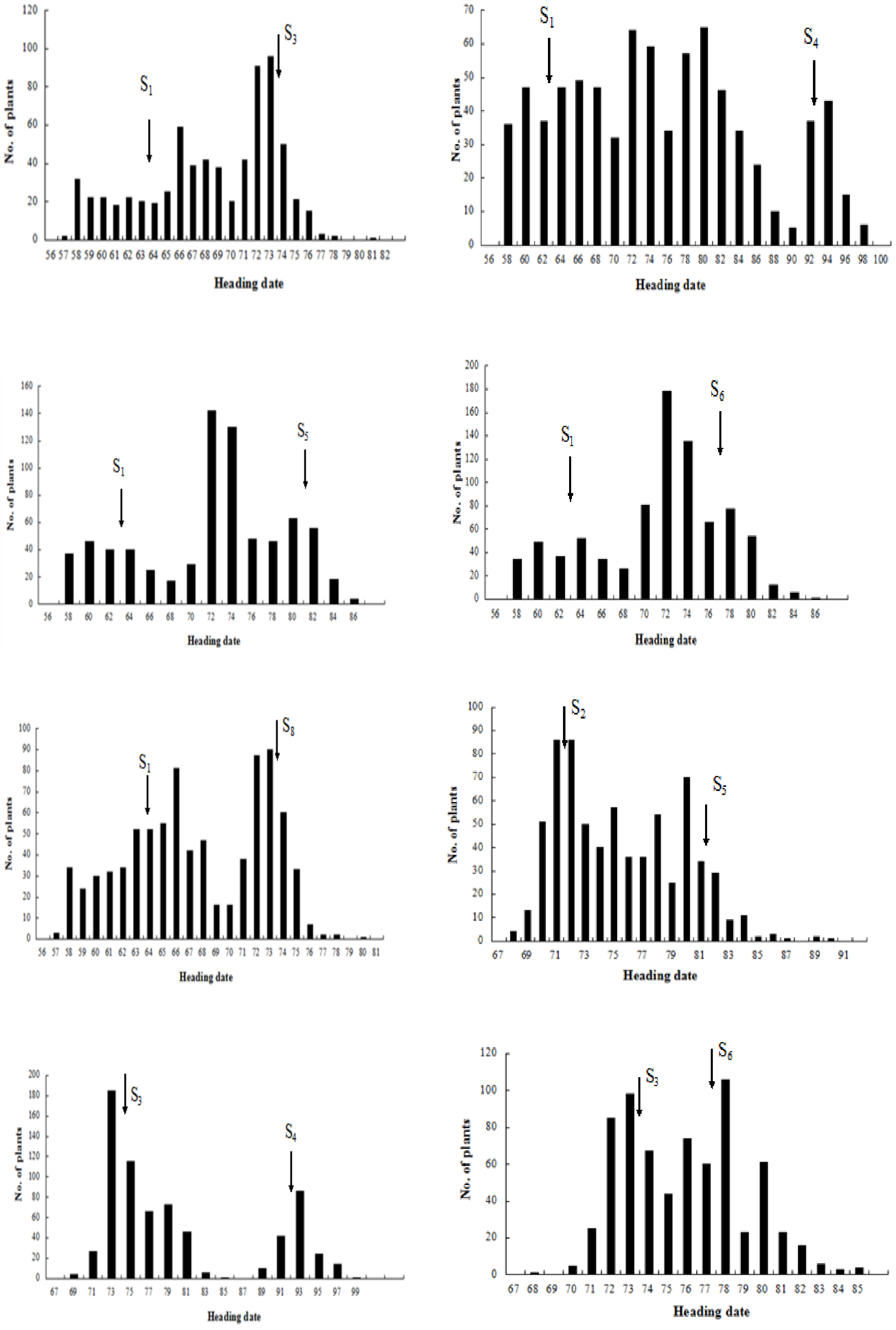

S$_1$
S$_3$
No. of plants
Heading date
S$_1$
S$_4$
No. of plants
Heading date
S$_1$
S$_5$
No. of plants
Heading date
S$_1$
S$_6$
No. of plants
Heading date
S$_1$
S$_8$
No. of plants
Heading date
S$_2$
S$_5$
No. of plants
Heading date
S$_3$
S$_4$
No. of plants
Heading date
S$_3$
S$_6$
No. of plants
Heading date

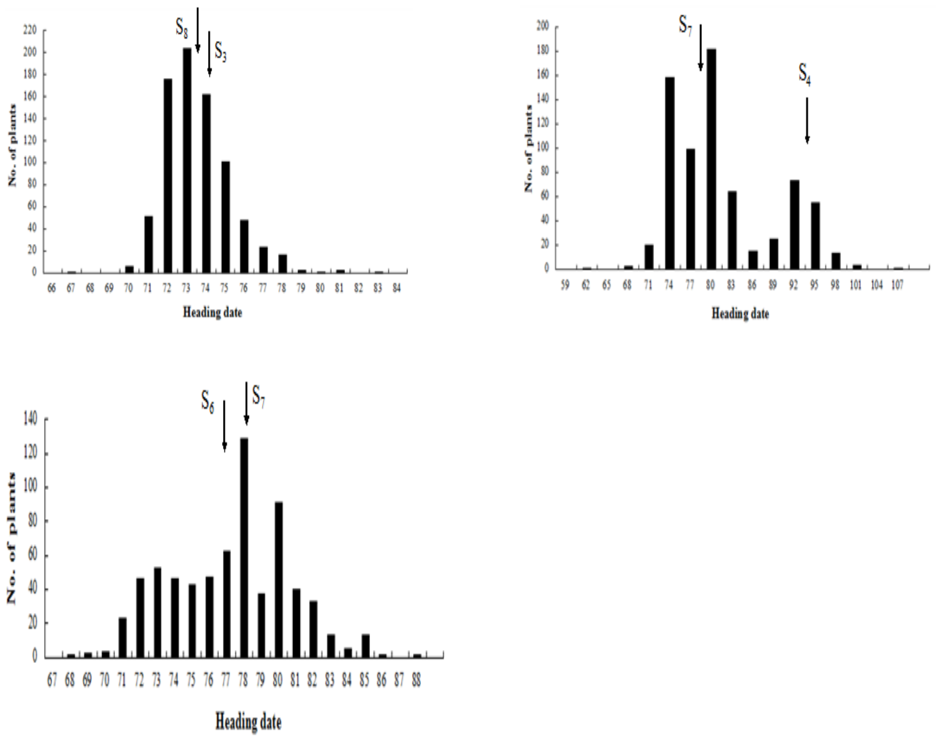

Supplementary Fig. 2 Heading date frequency distributions of F_2 populations from two single segment substitution lines in the late season of 2014. S_i represented single segment substitution line $SSSL_i$
